# Process Development for Adoptive Cell Therapy in Academia: A Pipeline for Clinical-Scale Manufacturing of Multiple TCR-T Cell Products

**DOI:** 10.3389/fimmu.2022.896242

**Published:** 2022-06-16

**Authors:** Daniela Nascimento Silva, Michael Chrobok, Giulia Rovesti, Katie Healy, Arnika Kathleen Wagner, Panagiota Maravelia, Francesca Gatto, Massimiliano Mazza, Lucia Mazzotti, Volker Lohmann, Margaret Sällberg Chen, Matti Sällberg, Marcus Buggert, Anna Pasetto

**Affiliations:** ^1^ Department of Laboratory Medicine, Karolinska Institutet, Stockholm, Sweden; ^2^ Division of Oncology, Laboratory of Cellular Therapy, Department of Medical and Surgical Sciences of Children and Adults, University of Modena and Reggio Emilia, Modena, Italy; ^3^ Clinical and Experimental Medicine PhD Program, University of Modena and Reggio Emilia, Modena, Italy; ^4^ Department of Dental Medicine, Karolinska Institutet, Stockholm, Sweden; ^5^ Department of Medicine Huddinge, Center for Infectious Medicine, Karolinska Institutet, Stockholm, Sweden; ^6^ Immunotherapy, Cell Therapy and Biobank (ITCB), IRCCS Istituto Romagnolo per lo Studio dei Tumori (IRST) “Dino Amadori”, Meldola, Italy; ^7^ Department of Infectious Diseases, Molecular Virology, University of Heidelberg, Heidelberg, Germany

**Keywords:** T cell modification, process development, pipeline, TCR-T cells manufacturing, clinical scale, academia

## Abstract

Cellular immunotherapies based on T cell receptor (TCR) transfer are promising approaches for the treatment of cancer and chronic viral infections. The discovery of novel receptors is expanding considerably; however, the clinical development of TCR-T cell therapies still lags. Here we provide a pipeline for process development and clinical-scale manufacturing of TCR-T cells in academia. We utilized two TCRs specific for hepatitis C virus (HCV) as models because of their marked differences in avidity and functional profile in TCR-redirected cells. With our clinical-scale pipeline, we reproduced the functional profile associated with each TCR. Moreover, the two TCR-T cell products demonstrated similar yield, purity, transduction efficiency as well as phenotype. The TCR-T cell products had a highly reproducible yield of over 1.4 × 10^9^ cells, with an average viability of 93%; 97.8–99% of cells were CD3+, of which 47.66 ± 2.02% were CD8+ T cells; the phenotype was markedly associated with central memory (CD62L+CD45RO+) for CD4+ (93.70 ± 5.23%) and CD8+ (94.26 ± 4.04%). The functional assessments in 2D and 3D cell culture assays showed that TCR-T cells mounted a polyfunctional response to the cognate HCV peptide target in tumor cell lines, including killing. Collectively, we report a solid strategy for the efficient large-scale manufacturing of TCR-T cells.

## Introduction

The clinical development of adoptive cell therapy (ACT) with *ex vivo* expanded genetically modified lymphocytes redirected towards different types of antigens is indeed rapidly and impressively moving from bench to bedside (1). In addition to chimeric antigen receptor (CAR)-T cells, T cell receptor (TCR)-T cells represent one of the major approaches of adoptive immunotherapy. TCRs recognize epitopes derived from the entire cellular proteome, including viral proteins, cancer germline antigens, tissue differentiation antigens, and mutation-derived antigens (cancer neoantigens); TCR-T cells exert their function through the canonical major histocompatibility complex (MHC)-dependent, TCR-mediated interaction and downstream signaling (2). ACT has already gained a consistent role in the treatment armamentarium of hematological malignancies, with some CAR-T cell-based products already approved in clinical practice (3–5). Furthermore, ACT is currently and tirelessly being exploited also in the more challenging context of solid tumors (6, 7). These therapeutic approaches together hold great promises for cancer therapy, although their translational efficiency is limited by the current manufacturing methods.

The manufacturing of genetically engineered T cells (CAR or TCR) is a multistep process consisting in the isolation of mononuclear cells, activation of T cells to favor susceptibility for the subsequent genetic (viral or non-viral) engineering, expansion of genetically modified cells, product harvesting, and cryopreservation (8). Good manufacturing practice (GMP)-compliant protocols have been described for this kind of purpose (9–14); however, several hands-on steps that might lead to a high risk of contamination, cell loss, and undesired mistakes are required. Moreover, the optimization of transduction conditions and expansion is a laborious process that can take up to 2 weeks to reach a clinically relevant cell number (15, 16), often impacting the T cell phenotype that might become exhausted and thus not optimal for clinical applications. Considering these aspects and the field of ACT rapidly expanding, the automation of the manufacturing process is an attractive alternative to minimize contaminations, simplify the procedure, increase the reproducibility (8, 17, 18), and enrich the final product for safe and clinically relevant cells with robust and persisting effector functions in patients (16).

Our group has previously isolated TCRs targeting hepatitis C virus (HCV) antigens and has demonstrated their effectiveness in redirecting T cells from healthy donors (HD) and chronically HCV-infected patients (19, 20). In the present study, we cloned two of the previously isolated TCRs into a lentiviral backbone vector—TCR-H4, specific for the HLA-A2-restricted epitope NS3_1073–1081_, and TCR-69, specific for the HLA-A2-restricted epitope NS5_1992–2000_, respectively—to be used as proof of principle in the process development of a novel clinical-grade manufacturing protocol that will definitely have future applications for the manufacturing of TCR-T cells targeting other antigens.

We performed process optimization from the research laboratory setting to clinical-grade manufacturing using the CliniMACS Prodigy system, a fully closed, automated, flexible, and user-defined platform that enables reproducible, scalable, and clinical-grade manufacturing of cell products with a single-use tubing system (8). Moreover, we evaluated the produced T cells from both phenotypic and functional points of view, highlighting an effort to scale up functionally active T cells by developing a fit-for-purpose platform to face the challenges of the emerging engineered therapy manufacturing.

## Materials and Methods

### Human PBMCs

Human peripheral blood mononuclear cells (PBMCs) from healthy blood donors (*n* = 3) and chronically infected patient (*n* = 1) were collected at the Karolinska University Hospital under informed consent and isolated using Ficoll-Hypaque (GE) density-gradient centrifugation. Ethical permission was obtained from the Regional Ethical Review Board (EPN) of the Karolinska Institutet.

### Cell Lines

Huh-7/Lunet cells, a hepatoma cell line used as target cells for cytotoxicity assays, were engineered with a subgenomic HCV replicon [Luc-ubi-neo Con1 as previously reported in (19)] and the firefly-luciferase gene as reporter gene, combined with a selectable marker (neomycin phosphotransferase), providing a convenient tool to study the role of antiviral therapies (21). Additionally, Huh-7/Lunet cells were engineered for ectopic HLA-A2 expression as previously described (22). Huh-7/Lunet cells referred to as Lunet HCV+/HLA-A2+ contain the HCV replicon and HLA-A2 gene and represent the real target for NS3 or NS5 redirected TCR-T cells. Huh-7/Lunet cells referred to as Lunet HCV+/HLA-A2- only contain the HCV replicon; thus, they represent a negative control for HLA-A2 specificity. Both Huh-7/Lunet cells were maintained in complete Dulbecco’s modified Eagle’s medium (DMEM) with the addition of puromycin (1 µg/ml) (GIBCO) and G418 (1 mg/ml) (GIBCO) in DMEMs (Thermofisher) supplemented with 10% fetal bovine serum (FBS, Sigma-Aldrich), 1x non-essential amino acids (Thermofisher), 2 mM L-glutamine (Thermofisher), and 100 mM HEPES (Thermofisher). The hepatoma cells were washed and reseeded 24 h in fresh medium before coculture.

T2 cells, commonly used as antigen-presenting cells, were used as another target cell line. T2 cells are a lymphoblastoid cell line deficient in transporter associated with antigen processing function and whose HLA class I protein can be easily loaded with exogenous peptides (23). T2 cells are an HLA-A2.1+ cell line used in the coculture assays in which NS3- or NS5-specific HLA-A2 peptides were loaded in different concentrations to evaluate the functionality of TCR-transduced T cells. The cells were grown in RPMI-1640 medium (Thermofisher) supplemented with 10% FBS (Sigma-Aldrich), 2 mM L-glutamine (Thermofisher), and 100 mM HEPES (Thermofisher).

### Synthetic Peptides

HCV NS3 genotype 1a peptide CINGVCWTV (aa 1,073–1,081), HCV NS5A (aa 1,992–2,000), and HIV peptide SLYNTVATL are referred to as NS3_1073-1081_, NS5A _1992-2000_, and HIV, respectively, and used to load T2 target cells pulsed with the indicated peptides for 2 h before their use in functionality assays. All peptides were synthesized by GenScript Biotech with purity >95%.

### Antibodies and Flow Cytometry Analysis

PBMCs and T cells were analyzed by flow cytometry (FACS) after staining with fluorochrome-conjugated monoclonal antibodies (mAb). The following antibodies were used: CD3 BUV650 (Biolegend), CD4 BV711 (Biolegend), CD8 BV570 (Biolegend), CD62L PE (BD Bioscience), CD45RO APC (BD Bioscience), anti-mouse TCRβ (mTCR) FITC (Invitrogen), CD25 PE-CF594 (BD Bioscience), CD69 PE (BD Bioscience), and PD-1 BB700 (BD Bioscience). Immune-cell composition and purity were determined using the following antibodies: CD3 BUV805 (BD bioscience), CD4 PE-Cy5.5 (Thermofisher), CD8 BV395 (BD Bioscience), CD14 BV711 (BD Bioscience), CD19 BV510 (BD Bioscience), and CD56 APC (BD Bioscience). Cell viability was determined by live and dead cell markers (Invitrogen) added freshly to each panel mix for 10 min at 4°C in the dark prior to staining with mAbs. The staining of cells was performed in 0.01M phosphate-buffered saline supplemented with 2% fetal bovine serum (FACS buffer) for 20 min at 4°C in the dark. To confirm the correct pairing and surface expression of the murine TCRs transferred into human T cells, APC-labeled HLAA*0201 dextramers refolded with HCV NS3_1073-1081_ or NS5_1999-2000_ (Immudex) were used.

For intracellular multicolor flow cytometry staining, TCR-transduced T cells were incubated for 6 h with the indicated stimuli. GolgiPlug and GolgiStop (BD Biosciences) were added during incubation. PMA and ionomycin (Sigma) were used as positive controls at 50 ng/ml and 1 ug/ml, respectively. The cells were then washed in FACS buffer and stained with the following: CD3 BUV650 (Biolegend), CD4 BV711 (Biolegend), CD8 BV570 (Biolegend), anti-mouse TCRβ (mTCR) FITC (Invitrogen), CD107a BUV395 (BD Biosciences), interleukin 2 (IL-2) PE (BD Biosciences), tumor necrosis factor α (TNFα) PE-Cy7 (BD Biosciences), and interferon γ (IFNγ) APC (BD Biosciences). A BD Cytofix/Cytoperm fixation/permeabilization kit was used after the staining procedure. Data acquisition was performed on a BD Symphony flow cytometer using FACS Diva Software (BD Biosciences). Data were analyzed using FlowJo 10 software.

### Production of Lentiviral Particles

Third-generation lentiviral vectors pRRLSIN.cPPT.MSCV.WPRE harboring HCV NS3- or NS5-specific TCRs driven by the murine stem cell virus (MSCV) U3 promoter were produced as previously described by (24); the parental pRRLSIN.cPPT.MSCV.WPRE plasmid encoding GFP was kindly provided by Dr. Steven Rosenberg, NCI, NIH (25). Lentiviral particles were manufactured at research-grade level by transient transfection of the lentiviral backbone vector together with three packaging helper plasmids—pMDLg/pRRE #12251, pRSV-Rev #12253, and pCMV-VSV-G #8454 (all from Addgene)—into HEK293FT cells (Thermofischer). Viral supernatants were harvested twice and filtered through a 0.45-μm filter followed by a concentration step with Lenti X concentrator (Takara Bio) following the manufacturer’s recommendation. The lentiviral particles were titrated in the presence of 8 µg/ml protamine sulfate (Sigma-Aldrich) on PBMCs, and the detection of transgene expression was performed 72 h after transduction by flow cytometry.

### Manufacturing of Redirected TCR-T Cells

Peripheral blood mononuclear cells were isolated from buffy coats by density gradient centrifugation and cryopreserved in 90% FBS and 10% dimethylsulfoxide (Sigma) at a density of 50 × 10^6^ white blood cells per milliliter. For freezing, the cells were stored at -80°C using a freezing container (CoolcellsCorning; Nalgene) for 24 h and transferred afterwards for long-term storage in liquid nitrogen. After thawing, the cells were immediately washed in GMP-grade TexMACS medium (Miltenyi) supplemented with 1 ug/ml DNase Pulmozyme (Roche) and reformulated in 40 ml TexMACS medium. The cell suspension was transferred into a 150-ml transfer bag (Miltenyi) and connected to the installed tubing set. Activation, transduction, and expansion of T cells were performed on the CliniMACS Prodigy using Tubing set TS520 (Miltenyi), and the T cell transduction (TCT) process was chosen as the template process.

On day 0, cultivation was initiated with 200 × 10^6^ PBMCs in a total volume of 70 ml of TexMACs GMP medium supplemented with premium-grade human recombinant IL-2, IL-15, and IL-21 cytokines (Miltenyi). TransAct CD3/CD28 reagent kit (Miltenyi) was used for 24-h activation as recommended by the manufacturer. On the following day, the cells were transduced with lentiviral particles, at multiplicity of infection (MOI) = 5, diluted in 10 ml of TexMACs GMP medium, and 10 μg/ml Vectofusin was allocated in a 20-ml transfer bag, which was attached to the CliniMACS Prodigy by sterile welding. The vector was then automatically transferred to the culture chamber, and the vector bag was further rinsed with 20 ml of the medium to bring to a total culture volume of 100 ml. Residual TransAct and lentiviral vector were removed by an automated culture wash 24 h after incubation. The cells were then expanded for 8 days before being harvested. Sampling was performed on days 4, 7, and 8 for in-process controls, including cell count, cell viability, transduction efficiency, and phenotyping. At the end of the expansion (day 8), the cells were automatically collected in 100 ml TexMACS medium and transferred into a sterile bag. For the final cellular product, the samples were taken for flow cytometry analysis to determine the purity, T cell phenotype, transduction efficiency, and safety quality controls. Small-scale experiments were performed with the same reagents in tissue culture plates to validate parameters such as cell expansion, cell viability, transgene expression, phenotype, and functionality of the transduced T cells.

### Safety Tests

Microbiological examination according to Ph. Eur. 2.6.27 was performed in the final cell product on day 8. Mycoplasma testing was performed by PCR provided by an analytical company (Eurofins, Germany). The endotoxin levels were measured using the Charles River Endosafe^®^-PTS™ system, according to the manufacturer’s instructions. All samples tested with the Endosafe system used 0.05–5.0 endotoxin unit/ml sensitivity cartridges provided by Charles River. BacT/ALERT^®^3D microbial detection system (BioMérieux) was used to detect fungal and aerobic and anaerobic bacterial contamination. G-band karyotyping was performed by a validated G-band assay at the laboratory of Clinical Genetics in Karolinska University Hospital. A total of 25 G-banded metaphases were analyzed.

### Droplet Digital PCR

Droplet digital PCR (ddPCR) was performed using the Bio-Rad QX200 system according to the manufacturer’s protocols (Bio-Rad). The ddPCR reaction mixture consisted of 10 μl of 2 × ddPCR master mix (Bio-Rad), 60 ng genomic DNA, and 20× primers/probes mix targeting RRE (FAM), a Rev response element sequence located upstream of the MSCV promoter in the integrated lentiviral genome (26). Primers and probes targeting the reference gene RPP30 (HEX) were added in a final volume of 21 μl in all reactions. The entire reaction mixture was loaded into a disposable plastic cartridge (Bio-Rad, USA) together with 70 μl of droplet generation oil (Bio-Rad) and placed into the droplet generator (Bio-Rad). After processing, the droplets generated from each sample were transferred into a 96-well PCR plate (Eppendorf). PCR amplification was carried out on a T100 Touch thermal cycler (Bio-Rad), and the acquired data were analyzed with QuantaSoft Analysis Pro software (Bio-Rad).

### Functional Analysis of Transduced T Cells

The TCR-transduced T cells were incubated in a U-bottom 96-well plate (Corning) with 1 × 10^5^ cells/well in a ratio of 1:1 with T2 cells loaded with different concentrations of NS3 and NS5 peptides (1,000, 100, 10, 0.5, 0.1, and 0 ng/ml). T2 cells loaded with HIV peptide (1,000 ng/ml) were included as a negative control. After 6 h of coculture, the intracellular content of human IL-2, TNFα, IFNγ, and CD107a in the TCR-transduced T cells was evaluated by intracellular multicolor flow cytometry staining.

### Cytokine Production Assay

The T cell products were rested overnight in a culture medium without cytokine. On the next day, the cells were cocultured overnight in 24-well tissue culture plates with 10^4^ cells of the indicated target cells in a ratio of E/T 1:1. PMA and ionomycin (Sigma) at 50 ng/ml and 1 ug/ml, respectively, were used as positive controls after 6 h of incubation. Supernatants were harvested after 24 h, and concentrations of human IL-2, IL-6, IL-10, IL-17, IL-4, TNFα, and IFNγ in the supernatants were analyzed by Milliplex map human high-sensitivity T cell magnetic bead panel assay (Merck) according to the manufacturer’s instructions.

### *In Vitro* 2D and 3D Cytotoxic Assays

For the 2D experiments, both Lunet HCV+/HLA-A2+ and Lunet HCV+/HLA-A2- cells were cocultured with transduced or mock non-transduced T cells in different E/T ratios (1:1, 0.1:1, and 0.01:1). Following 24 h of coincubation, the medium was replaced with luciferin solution (Promega) prior to imaging with a charge-coupled device camera. Signals from bioluminescent Lunet HCV+/HLA-A2+ and Lunet HCV+/HLA-A2- cells were analyzed with the Living Image Software, version 4.2, and IVIS Spectrum instrument (Caliper LifeSciences). The aspartate transaminase (AST) levels in supernatants were quantified by a validated AST assay at the Clinical Chemistry Laboratory of the Karolinska University Hospital, Solna, Sweden, using the Modular P apparatus (Roche Diagnostics, Mannheim, Germany).

For the 3D experiments, both Lunet HCV+/HLA-A2+ and Lunet HCV+/HLA-A2- cells were stained in 1 µM Calcein-AM (Invitrogen) diluted in fresh medium for 30 min at 37°C prior to spheroid formation by seeding 30,000 cells per well in a Costar ultra-low attachment round-bottom 96-well plate (Corning). For the 3D assays, cells were cultured in a humidified 37°C, 5% CO_2_ incubator for 48 h for the formation of a single spheroid in each well. For the 3D tumor cell death analysis, spheroids were stained with Annexin V green reagent (1:200) (Sartorius) for 15 min at room temperature prior to the coculture experiments. The TCR-transduced T cells were seeded in a ratio of E/T at 1:1, and cell viability and cell death evaluations were performed by the acquisition of imaging green fluorescence across time using the Incucyte S3 system (Sartorius). Single pictures of each well were acquired every hour for 5 days and analyzed using Incucyte software, version 2019B.

### Statistical Analysis

Data are displayed as the mean ± SEM analyzed with Prism version 8.0 (GraphPad Software). Unpaired Student’s *t*-test was used for the comparisons between two groups. Two-way ANOVA was used to compare multiple groups. A *p*-value <0.05 was considered to be significant.

## Results

### Small-Scale Generation and Functional Characterization of HCV-Specific TCR-T Cells

**Figure 1:**
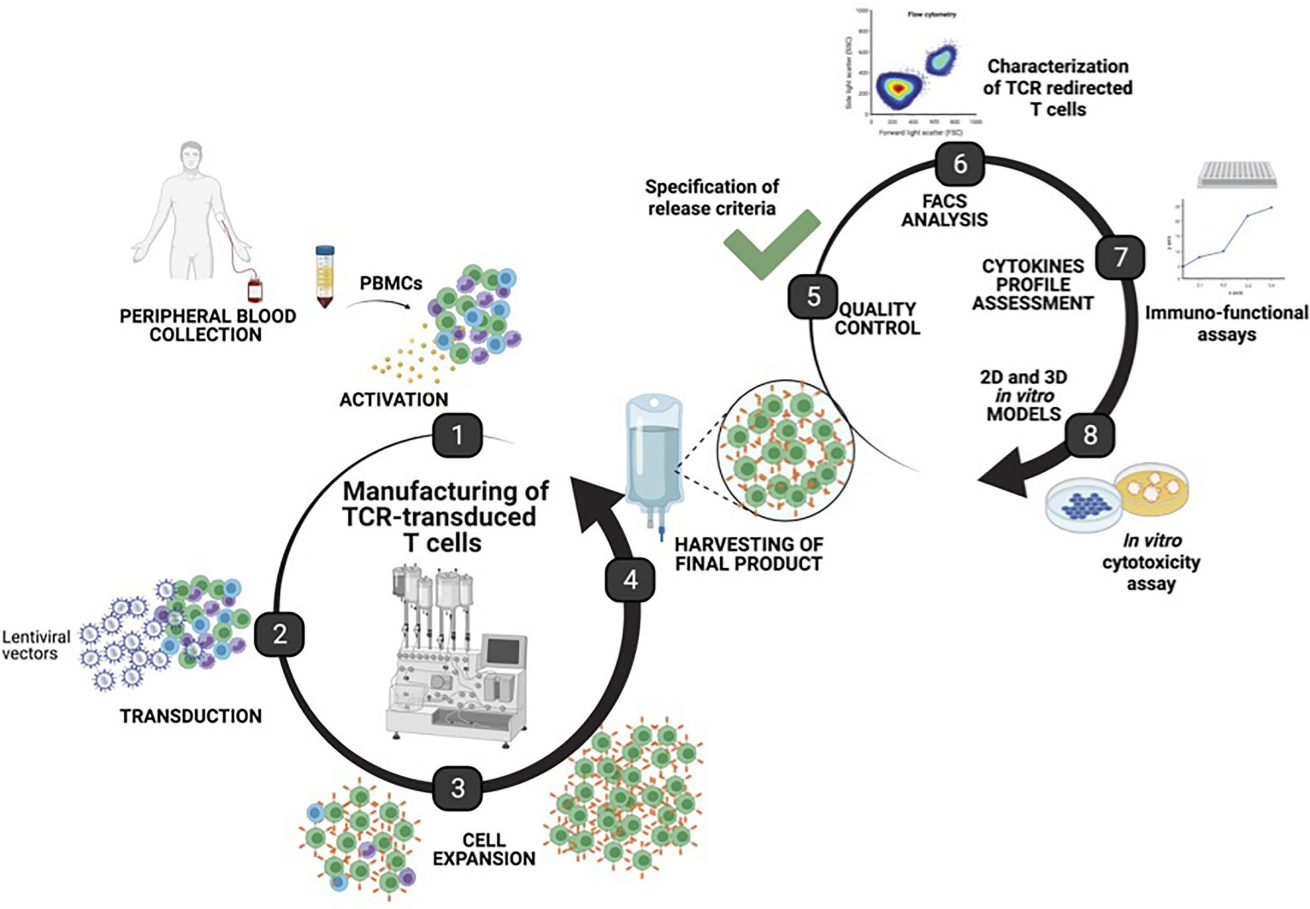
Manufacturing of T cell receptor (TCR)-T cells using the CliniMACS Prodigy. Graphical representation of the manufacturing steps for TCR-T cells using the automated, closed CliniMACS Prodigy platform (steps 1–4). Frozen peripheral blood mononuclear cells were obtained from healthy donor buffy coat and used as starting material for the activation, transduction, and expansion of T cells under optimized conditions. After 8 days of expansion, samples from the harvested final products were taken for safety quality controls, and characterization of TCR-T cells was performed to determine the purity, T cell phenotype, transduction efficiency, functionality, and cytotoxic potential as assessed by 2D and 3D *in vitro* models (created with BioRender.com).

We developed a manufacturing procedure (**Figure 1**) for the expansion and transduction of human T cells with a 3rd-generation lentiviral vector encoding for two unique HCV-specific T cell receptors that recognize the HLA-A2-restricted CTL epitopes—the NS3_1073_ and NS5_1992_ of HCV (20).

To test the efficiency of these reagents for the stable transduction of human T cells as well as to examine if PBMCs from chronic patients could be redirected with the same efficiency, small-scale experiments were carried out with PBMCs from healthy donors (HD1, HD2, and HD3) and from a chronically HCV-infected patient (KS6) as described above. The cell expansion, phenotype, transduction efficiency and functionality of TCR-redirected T cells were evaluated. Lentiviral particles encoding the NS3- and NS5-specific TCRs were used, at 24 h post-activation, to transduce T cells from HLA-A2 healthy donors and a chronically HCV-infected patient with a MOI of 5.

**Figure 2:**
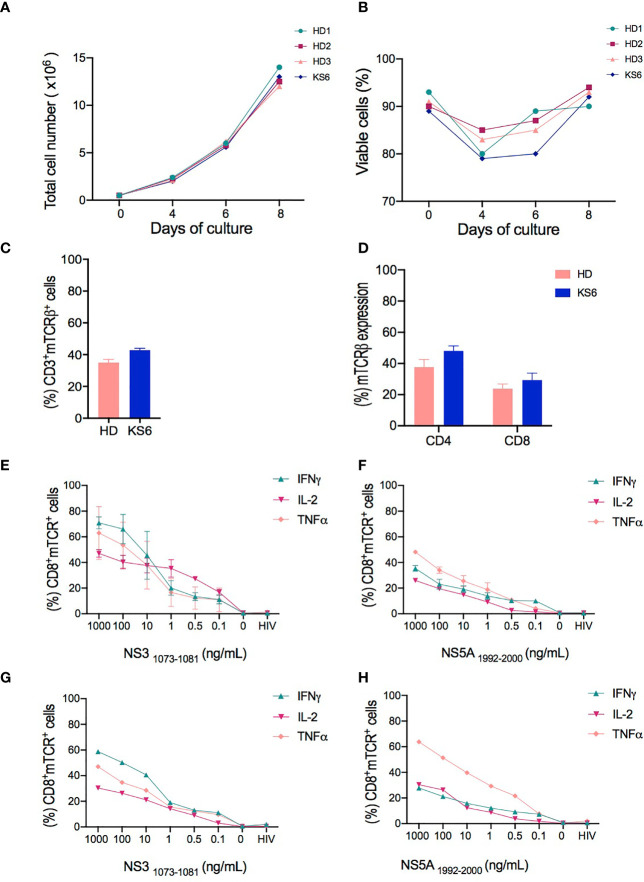
Characterization of T cell receptor (TCR)-T cells produced in small-scale experiments. **(A, B)** Comparison of the total cell number and viability of cell products from healthy donors (HD1-3) and chronically hepatitis C virus (HCV)-infected patient (KS6) performed on days 0, 4, 7, and 8 of cell expansion. **(C)** Transduction efficiency of redirected TCR-T cells represented by the percentage of mTCR expression in CD3+ cells from healthy donors and HCV-infected patient (KS6) after 8 days of expansion. **(D)** Percentage frequencies of mTCR expression in CD4+ and CD8+ T cells following T cell expansion. HCV-specific responses assessed by flow cytometry analysis for the intracellular content of IFNγ, TNFα, and IL-2 in CD8+ transduced cells following 6 h of coculture of NS3_1073–1081_ or NS5_1992–2000_ peptide-loaded T2 cells with redirected TCR-T cells from healthy donors **(E, F)** and chronically HCV-infected patient **(G, H)**. HIV peptide-loaded T2 cells and non-stimulated T cells were included as negative controls in the coculture experiments performed in triplicate wells.

We compared the absolute number and cell viability of transduced cells from healthy donors and the chronically HCV-infected patient after 8 days of expansion. As illustrated in **Figures 2A, B**, no difference in total cell number (12.50 × 10^6^ ± 0.42 × 10^6^) and cell viability (92.50% ± 0.85) of the expanded T cells was observed between the healthy donors and the HCV-infected patient. A drop in cell viability was observed in both cell sources after 4 days of expansion due to the selective T cell survival after activation by Transact (27). A consistent growth pattern was observed among the four samples tested regardless of whether the samples had been obtained from healthy donors or the HCV-infected patient.

Furthermore, we analyzed the surface expression of the introduced TCRs. We used a lentiviral vector encoding NS3- and NS5-specific TCRs engineered with mouse TCRβ constant domain, which avoids endogenous TCR mispairing, as previously described (28), and enables the detection of the exogenous TCRs through the use of antibodies against the mouse Vβ chain. The flow cytometry analysis revealed that 35% ± 2.03 of mTCRβ were detected in live CD3+ healthy donor T cells and 42.9% ± 1.1 in the chronically HCV-infected patient (**Figure 2C**). The majority of transduced cells were CD4^+^ T cells both in healthy donors (37.6% ± 2.00) and in the HCV-infected patient (48% ± 2.30) compared to 23.75% ± 1.27 and 29.35% ± 3.1 CD8^+^ T cells in healthy donors and the HCV-infected patient, respectively (**Figure 2D**).

We next assessed the HCV-specific responses of TCR-T cells following coculture against NS3_1073–1081_ or NS5_1992–2000_ peptide-loaded T2 cells. The measurements of IFNγ, TNFα, and IL-2 expression were performed at the intracellular level in CD8+ cells and evaluated by flow cytometry. We found that both NS3- and NS5-redirected T cells from the healthy donors (**Figures 2E, F**) and the HCV-infected patient (**Figures 2G, H**) secreted more than one cytokine, such as IFNγ, TNFα, or IL-2, in a dose-dependent manner. However, NS3-redirected T cells secreted mostly IFNγ and TNFα, whereas NS5-redirected T cells mainly produced TNFα. Our findings confirm that NS3-specific TCR-T cells were superior IFNγ producers compared to NS5-specific TCR-T cells, which is consistent with the previously described results of T cells engineered by retroviral transduction or transiently transfected with mRNA (20, 29). Given the stable expression of the TCRs and the confirmation of their functional attributes, we proceeded with the optimization of large-scale manufacturing.

### Large-Scale Manufacturing Outline

To develop and assess a GMP-compatible T cell product, we optimized an automated protocol to produce TCR-T cells, carrying out three independent runs (runs 1–3). All runs were performed using the CliniMACS Prodigy Tubing Set TS 520, which was installed on the CliniMACS Prodigy. Each individual run included PBMC isolation by Ficoll gradient followed by the indicated steps outlined in **Table 1**. During all runs, samples for quality controls were taken on days 0, 4, 7, and 8 to monitor the quality and quantity of cells. Parameters such as sterility, viability, identity, purity, karyotyping, transduction efficiency, and potency were assessed to validate the clinical grade compliance of the final product. All analyzed samples met the acceptance criteria summarized in **Table 2**. Additionally, the immunophenotype of TCR-T cells was determined by flow cytometry, and potency assays were performed to assess the phenotype and the functionality of TCR-T cells. An illustration of the different steps for TCR-T cell manufacturing and the quality tests conducted along the process is shown in **Figure 1**.

**Table 1:** T cell manufacturing methodology.

Steps	Methods	Timepoint
T cell activation	Polymeric biodegradable CD3/CD28 incorporating nanomatrix	Days 1 and 2
T cell stimulation	IL-2, IL-15, and IL-21 in the culture medium	From day 1 onwards
Transduction with lentiviral vector	Vectofusin used to enhance transduction	Days 2 and 3
T cell expansion	CliniMACS Prodigy	Days 3 and 4 onwards
T cell harvest and cryopreservation	Passive freezing -80°C (1°C/min)	Day 8
Quality assurance control and releasing testing	In-process and end of process controls are taken to ensure that the product complies with the release criteria specifications	Day 8 onwards

**Table 2:** Quality control of TCR-T cells.

Testing	Criteria	Specification	Acceptance criteria
**Release**	Sterility	Mycoplasma (PCR) Endotoxin Microbial growth	Negative <2 EU/ml Sterile from bacteria/fungi
	Viability	Nucleocounter	>70%
	Identity	Flow cytometry	>90% T cells
	Purity	Flow cytometry	<10%
	Transduction efficiency	Flow cytometry	>20%
	Karyotype	G-band	46 XX, XY
	Number of transgene copies/cell	ddPCR	<5 copies/cell
	Potency	ELISA	IFN-γ secretion

### Automated T-Cell Transduction and Expansion Using CliniMACS Prodigy

To validate our protocol on the CliniMACS Prodigy, large-scale experiments were performed using cryopreserved PBMCs from healthy donors (*n* = 3) previously tested in small-scale experiments. Transduction was performed with lentiviral particles harboring NS3- (*n* = 2) and NS5-specific TCRs (*n* = 1) added by sterile welding of the supply bag after 24 h of activation with TransAct reagent (Miltenyi). The feeding conditions were optimized to support 8 days of cell expansion by increasing the volume of media exchange. Parameters such as total cell number, cell viability, T cell activation, transduction efficiency, purity, and T cell exhaustion were evaluated.

**Figure 3:**
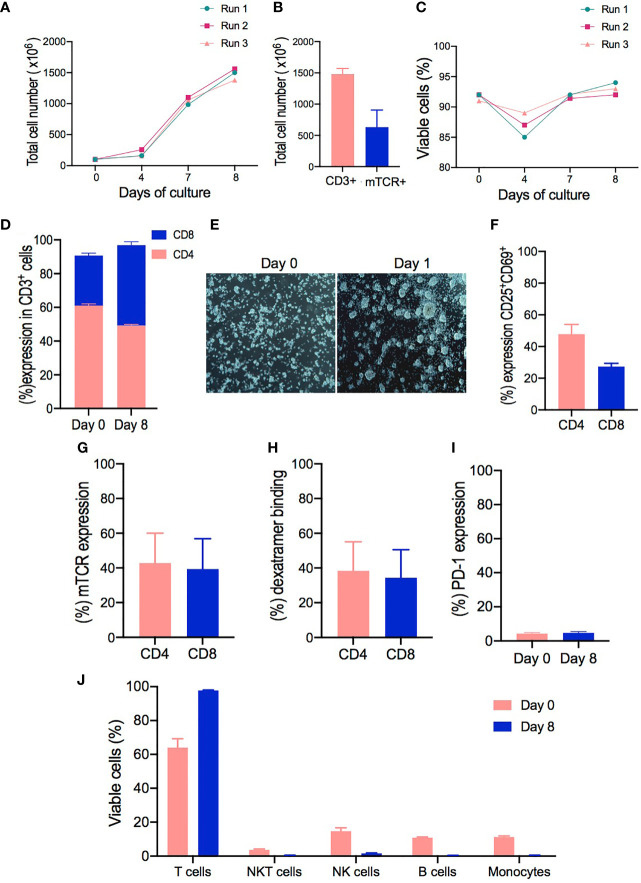
Clinical-grade manufacturing of T cell receptor (TCR)-T cells using the CliniMACS Prodigy. **(A–C)** Total cell numbers and cell viability of cell products from healthy donors (HD1-3) performed on days 0, 4, 7, and 8 of cell expansion. **(D)** CD4 and CD8 T cell ratios at the start of culture (day 0) compared to the end of culture (day 8). **(E)** Representative pictures from cell cultures taken with the internal camera of the CliniMACS Prodigy on day 0 and at 24 h after activation (day 1) under static culture conditions. **(F)** Activation status of CD4+ and CD8+ T cells after 24 h of activation as evaluated by flow cytometry analysis for the co-expression of CD25 and CD69 surface markers. **(G)** Transduction efficiency of redirected TCR-T cells represented by the percentage of mTCR expression in CD4+ and CD8+ cells on day 8. **(H)** Percentage frequencies of dextramer-specific binding in CD4+ and CD8+ transduced T cells. **(I)** Comparison of PD-1 expression evaluated on cells from starting material (day 0) and expanded transduced T cells (day 8). **(J)** Cell composition of starting material on day 0 and expanded final product on day 8 as assessed by flow cytometry to identify the percentage frequencies of lymphocyte T cells (CD3+), monocytes (CD14+), B cells (CD20+), NK cells (CD3-CD56+), and NKT cells (CD3+CD56+). The results are shown as mean ± SD for three independent healthy donors (*n* = 3).

The expansion fold of lymphocytes in the CliniMACS Prodigy was approximately 15-fold change and was comparable to the expansion achieved in small-scale experiments from the same donors. The absolute number of viable cells was monitored from day 0 to day 8 of culture, with most of the cell expansion occurring between days 4, 7, and 8. This resulted in an average yield of 1.48 × 10^9^ cells ± 52.91 total lymphocytes (**Figure 3A**) and 6.32 × 10^8^ transduced mTCR+ cells (**Figure 3B**) from the starting number of 1.00 × 10^8^ T cells. No significant reduction in cell number was observed during the cell expansion, as observed for small-scale, and the cell viability decreased from day 0 to day 4 of culture.

Since a clinically relevant dose has not been determined yet for this type of products, we calculated that, with our protocol, all tested samples expanded to a range that would be sufficient to perform an initial phase I/dose escalation trial similar to the one performed by Meng *et al.* in patients affected with hepatitis B-related hepatocellular carcinoma (30). In this trial, the patients were given a dose range starting from 1 × 10^4^ CD8+Vβ+ T cells/kg up to a maximum dose of 5 × 10^6^ CD8+Vβ+ T cells/kg. This range appears to be achievable with our manufacturing process, showing a median of 1.48 × 10^9^ cells ± 52.91 total lymphocytes with 93% ± 1 viability (range, 87–93%) (**Figure 3C**), being 279.3 × 10^6^ ± 101.2 mTCR+CD4+ cells and 290.6 × 10^6^ ± 113.7 mTCR+ CD8+ cells.

Interestingly, the expansion conditions led to a selective increase of CD8+ cells in all samples, decreasing the average CD4:CD8 ratio from 2.06 on day 0 to 1.03 by the end of culture. In addition, cells expanded in the CliniMACS Prodigy with our protocol had a greater percentage of CD8+ T cells in the final product (47.66% ± 2.02) compared to cells from the starting material on day 0 (29.66% ± 1.45) (**Figure 3D**). Thus, the NS3- and NS5-specific TCR-T cells from healthy donor samples had a very consistent, viable, and robust expansion especially for the CD8+ compartment.

We next evaluated the level of cell activation. Most T cell manufacturing protocols use either soluble or magnetic bead-bound anti-CD3/CD28 antibodies to activate the T cells before transduction (31). In contrast, the automated TCT protocol for the CliniMACS Prodigy adopts TransAct, a soluble, colloidal reagent with covalently attached anti-CD3/CD28 antibodies (32), which can be removed from the culture by washing the cells and obviates the need for a bead removal step. Here we evaluated the suitability of TransAct to activate T cells from a heterogenous cell source (PBMCs) prior to transduction. Clumping cells were observed under the integrated microscope from the CliniMACS Prodigy on day 1, indicating a cell culture morphology associated with activated and proliferative cells after 24 h of activation compared to non-activated cells on day 0 (**Figure 3E**). Subsequently, the activated T cells were assessed by flow cytometry, showing the upregulation of CD25 and CD69 surface molecules in CD4+ and CD8+ T cells. Both CD4+ and CD8+ T cells upregulated CD25 and CD69 expression, although CD4+ cells were more efficiently activated (47.6% ± 3.56) compared to CD8+ cells (27.36% ± 1.15) (**Figure 3F**).

In addition, flow cytometry was used to monitor the transduction efficiency and changes in cell populations within the culture over time. In three scaled productions, the multiplicity of infection of the samples was 5, leading to a vector copy number of 1.09–1.52 per TCR-transduced T cell. The transduction efficiency was evaluated by the expression of TCR mouse Vβ chain, which averaged 42.8% ± 9.96 in CD4+ T cells (range, 23.3–56.1%) compared to 39.33% ± 10.11 (range, 19.4–43.9%) in CD8+ T cells (**Figure 3G**). Moreover, specific HCV peptides/MHC dextramer complexes for each TCR were used to confirm the appropriate pairing of the transduced TCR. NS3 and NS5/HLA-A2 dextramer staining revealed that both TCRs bound their respective dextramer in CD4+ (38.36% ± 9.67) and CD8+ cells (34.33 ± 9.33), indicating that they are stably expressed on the cell surface and retain their specificity for their cognate ligand (**Figure 3H**).

The expression of PD-1 was evaluated after *ex vivo* activation and expansion, as this marker could give an insight on the activation/exhaustion status of the cells (33). The expression of PD-1 did not differ significantly from cells in the starting material (day 0) compared to the final product on day 8 (**Figure 3I**). Product purity represents one of the major concerns for the safety and the outcome of patients (34). The cellular composition of the TCR-T cell products was analyzed by flow cytometry for the presence of CD3+ T cells, CD14+ monocytes, CD19+ B cells, CD3-CD56+ NK cells, and CD3+CD56+ NKT cells at the harvest step on day 8. As shown in **Figure 3J**, there was little to no contamination of B cells (mean, 0.38%; range, 0.36–0.45%), monocytes (mean, 0.46%; range, 0.41–0.52%), NK cells (mean, 1.65%; range, 1.45–1.9%), and NKT cells (mean, 0.4%; range, 0.3–0.5%), and a mean CD3+ T cell purity of 98% (range, 97.8–99%) was achieved in 3 runs. Overall, these results confirmed the potential of our protocol to support the expansion of T cells with high viability and purity and low percentage of exhausted cells.

### Phenotypic and Functional Characterization of Large-Scale-Produced Cells

**Figure 4:**
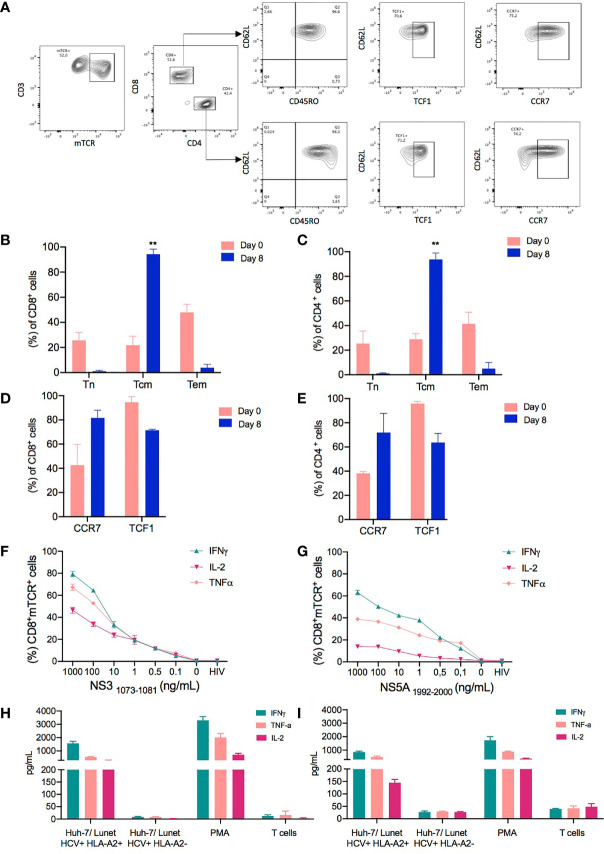
Phenotype and functionality of T cell receptor (TCR)-T cells produced in a large scale. The phenotypic characteristics of cells expanded in the CliniMACS Prodigy was assessed by flow cytometry to identify the percentage of memory T cell markers in TCR-T cells. **(A)** Representative FACS plots showing the gating strategy for the evaluation of memory T cell populations. The memory T cell populations were analyzed by gating on live lymphocytes based on their FSC-A/SSC-A profile and excluding any doublets. Subsequently, CD4+ and CD8+ mTCR+ transduced cells were analyzed in regard to their T cell subpopulations as assessed by the expression of CD45RO and CD62L. Besides the Tcm subpopulation (CD62L+ CD45RO+), the CCR7 and TCF1 expressions were evaluated in the CD62L+ population. The subpopulations were defined as follows: naïve T cells, CD62L+CD45RO-; central memory T cells (Tcm), CD45RO+CD62L+; and effector memory T cells (Tem), CD62-CD45RO+. **(B, C)** Column graphs showing the relative frequencies of the indicated subpopulations in CD8+ **(B)** and CD4+ T cell subsets **(C)** for each time point analyzed (days 0 and 8). **(D, E)** Comparison of CCR7 and TCF1 expressions evaluated on cells from the starting material (day 0) and expanded transduced T cells on day 8. **(F, G)** Hepatitis C virus (HCV)-specific responses as assessed by flow cytometry analysis for the intracellular content of IFNγ, TNFα, and IL-2 in CD8+ transduced T cells following 6 h of coculture of NS3_1073–1081_ or NS5_1992–2000_ peptide-loaded T2 cells with the indicated NS3- or NS5-specific TCR-T cells manufactured in CliniMacs Prodigy. HIV peptide-loaded T2 cells and non-stimulated T cells were included as negative controls in the coculture experiments performed in triplicate wells. **(H, I)** Cytokine production of TCR-T cells when cocultured with Huh-7/Lunet HCV replicon target cells as measured by Milliplex map human high-sensitivity kit. Huh-7/Lunet HCV replicon cells that were not engineered to express HLA-A2 (Lunet HCV+/HLA-A2-) as well as non-stimulated T cells were used as negative controls in the experiments performed with technical triplicates. The results are shown as mean ± SD for three independent healthy donors. ** is p value ≤ 0.01.

Immunophenotyping of cells expanded in the CliniMACS Prodigy was performed to further characterize the T cell subpopulations in the final product. In principle, engineered T cells should be long-lived to ensure protection for an extended period of time; however, the survival and self-renewal rates are heterogeneous among different T cell subpopulations (35). Naïve T cells (Tn) can be distinguished from memory T cells, which can be further subdivided into central memory (Tcm) and effector memory T cells (Tem) (17). As previous studies have emphasized, the induction of a Tcm phenotype in genetically modified T cells is desirable for improved self-renewal, formation of memory, effector differentiation, and lowered cytokine release syndrome (16). To evaluate the phenotypic characteristics of T cells from the starting material and the generated TCR-T cells in the final product, viable CD4+ and CD8+ transduced T cells were identified based on the expression of CD62L and CD45RO markers (**Figure 4A**). Tcm cells (CD62L+ CD45RO+) were the most prevalent subpopulation in both CD4+ (93.7% ± 5.23) and CD8+ (94.26% ± 4.04) subsets in the final product compared to the starting material on day 0, whereas Tem (CD62- CD45RO+) represented around 5.07% ± 4.9 in CD4+ cells and 3.9% ± 2.65 in CD8+ cells. As expected, naïve T cells (CD62L+CD45RO-) were present in the starting material (25.5% ± 10.24 in CD4+ and 25.6% ± 6.3 in CD8+), but their frequency diminished in the final product (1.14% ± 0.5 in CD4+ and 1.05 ± 0.7 in CD8+) for all the analyzed runs (**Figures 4B, C**).

To determine whether the CD62L+CD45RO+ T cells generated in our protocol were truly Tcm cells or had acquired memory traits, we performed further phenotypical analyses using established markers and a transcription factor associated with T memory cells and stemness, such as C-C chemokine receptor type 7 (CCR7) and transcription factor 7 that, in humans, encodes for the T cell factor 1 (TCF1) protein (36). CCR7 expression was upregulated in the final product compared to the starting material in both CD4+ (38.21% ± 1.5 on day 0 and 71.95% ± 15.75 on day 8) and CD8+ transduced T cells (42.65% ± 17.15 on day 0 and 81.6 ± 6.4 on day 8) (**Figures 4D, E**). Importantly, the frequency of TCF1 was maintained in CD4+ and CD8+ transduced cells after 8 days of expansion compared to the cells on day 0. Thus, we hypothesized that the culture conditions favored the expansion of T cell populations skewed towards an early differentiated memory phenotype.

To assess the functional capabilities of TCR-redirected T cells, we stimulated both NS3-specific and NS5-specific transduced T cells with T2 target cells pulsed with titrated amounts of NS3_1073-1081_ and NS5_1999-2000_ peptides for 6 h. As shown in **Figures 4F, G**, CD8+ NS3- and NS55-specific TCR-T cells cocultured with either NS3 or NS5 peptide-loaded T2 cells predominantly expressed IFNγ, followed by TNFα and IL-2, in a dose dependent manner at the intracellular level. Although NS3- and NS5-specific TCR-T cells expressed the same cytokine profiles, the levels of expression of IFNγ, TNFα, and IL-2 is reduced in NS5-specific T cells when evaluated intracellularly. The functional capabilities of CD4+ cells were also evaluated; however, the levels of expression of IFNγ, TNFα, and IL-2 were reduced in this population, although both TCRs, NS3 and NS5, are CD8 independent (**Supplementary Figure S1**).

Considering the distinct functional profiles of NS3- and NS5-specific TCRs previously described (20, 29), we next examined their cytokine production when targeting Huh-7/Lunet replicon cells that harbor the subgenomic HCV replicon maintained at constant levels of HCV replication. In addition, Huh-7/Lunet HCV replicon cells were stably transduced with a lentiviral vector expressing HLA-A2 and a selectable marker encoding the puromycin resistance gene as previously described (Lunet HCV+/HLA-A2+) (22). Huh-7/Lunet HCV replicon cells that were not engineered to express HLA-A2 (Lunet HCV+/HLA-A2-) as well as mock non-transduced cells were used as negative controls in the experiments.

After 24 h of incubation, supernatants from cocultures at 1:1 E/T ratio of NS3- and NS5-specific TCR-T cells and Hu7/Lunet cells were used to quantify the cytokines secreted by activated T cells. Both NS3- and NS5-specific TCR-T cells secreted pro-inflammatory cytokines associated with an effector/T helper 1 (TH1) phenotype such as IFNγ, TNFα, and IL-2 in response to their target antigen, whereas cytokine secretion was hardly detectable upon coculture with control Lunet HCV+/HLA-A2- or in the absence of a stimulus (**Figures 4H, I**). Although all cell products were able to produce inflammatory cytokines, higher levels of IFNγ, TNFα, and IL-2 were detected in the supernatants of stimulated NS3-specific TCR-T cells compared to NS5-specific TCR-T cells. Importantly, the TCR-T cells generated with our protocol produced lower levels of the regulatory cytokine IL-10 or cytokines associated with either a TH2 or TH17 phenotype, such as IL-4, IL-6, and IL-17, respectively (**Supplementary Figure S2**) (37). These results indicate that the TCR-T cells generated with our protocol have an early differentiated memory phenotype along with functionality compatible with an effector/TH1 phenotype.

### Potency of TCR-Redirected T Cells Assessed in 2D and 3D *In Vitro* Models

**Figure 5:**
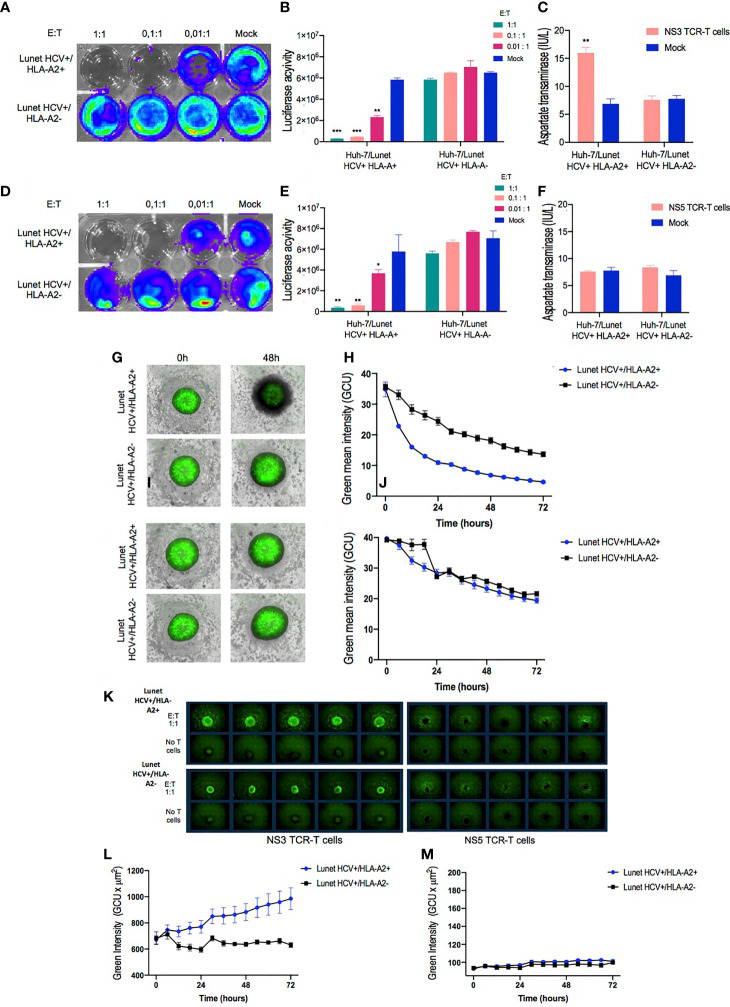
*In vitro* functional validation of TCR-T cells. NS3- and NS5-specific T cell receptor (TCR)-T cells demonstrate HLA-A2-restricted antiviral response against Huh-7/Lunet HCV replicon target cells in 2D cell culture model. Representative images of bioluminescence signal captured by IVIS Spectrum instrument from Huh7/Lunet HCV replicon cells after 24 h of cocultivation with NS3-specific TCR-T cells **(A)** or NS5-specific TCR-T cells **(D)** in the indicated E/T ratios (1:1, 0.1:1, and 0.01:1). Column bar graphs showing the Luciferase activity analyzed with Living Image Software in Huh-7/Lunet HCV+ cells cocultured with NS3-specific TCR-T cells **(B)** or NS5-specific TCR-T cells **(E)**. Quantification of aspartate transaminase levels in the supernatants from 24-h coculture of Huh-7/Lunet HCV replicon cells with NS3-specific TCR-T cells **(C)** or NS5-specific TCR-T cells **(F)** at 1:1 E/T ratio. 3D Spheroid Killing Assay was performed to assess the cytotoxicity of NS3-specific TCR-T cells and NS5-specific TCR-T cells. Representative images of Huh-7/Lunet HCV replicon cell spheroids stained with Calcein cell-permeant green dye following the addition of NS3-specific TCR-T cells **(G)** or NS5-specific TCR-T cells **(I)** at basal time (0 h) and 48 h of coincubation. **(H)** Graph showing the green mean intensity from viable cells in the spheroids after 72 h of coculture with NS3-specific TCR-T cells or NS5-specific TCR-T cells **(J)** analyzed by the Incucyte live imaging system. **(K)** Representative images of Huh-7/Lunet HCV+ cell spheroids stained with Annexin V green dye cocultured or not with te indicated NS3-specific TCR-T cells or NS5-specific TCR-T cells and monitored by 72 h using the Incucyte live imaging system. **(L)** Graph showing the green intensity measurements of Annexin V fluorescent binding cells following the addition of NS3-specific TCR-T cells or NS5-specific TCR-T cells **(M)** monitored by 72 h Huh-7/Lunet HCV replicon cells that were not engineered to express HLA-A2 (Lunet HCV+/HLA-A2-) as well as mock non-transduced T cells were used as negative controls in 2D experiments. The results are shown as mean ± SD (***p ≤ 0.001) for technical triplicates performed in the 2D experiments or five replicates in the 3D Spheroid Killing Assay. * is *p* value ≤ 0.05; ** is *p* value ≤ 0.01; ***p ≤ 0.001.

To further verify the efficiency of our protocol in generating functional TCR-T cells after a large-scale production, a cytotoxicity assay was implemented against Lunet HCV+ replicon cells previously engineered to express the firefly luciferase under the HCV replicase activity (19, 22). Luciferase expression was analyzed with the bioluminescence imaging system (IVIS Spectrum) using a high-dimensional charge-coupled device camera. The engineered Lunet HCV+ cells, together with the TCR-T cells, were incubated at different E/T ratios (1:1, 0.1:1, and 0.01:1), and the intensity of bioluminescence was measured. As illustrated in **Figure 5A**, images taken 24 h after coincubation revealed that NS3-specific TCR-T cells could reduce the bioluminescence intensity of Lunet HCV+/HLA-A2+ in a dose-dependent manner (**Figures 5A, B**). On the other hand, NS5-specfic TCR-T cells were less effective down to 0.001 E/T ratio, although a reduction of bioluminescence could still be observed in the experiments (**Figures 5D, E**). No significant inhibition of HCV replication was observed when NS3- or NS5-specific TCR-T cells or mock non-transduced T cells were cocultured with Lunet HCV+/HLA-A2-.

Furthermore, supernatants from cocultures at 1:1 E/T ratio were used to quantify AST secreted by Huh-7/Lunet HCV replicon cells. A significant increase of hepatocellular AST was detected in the coculture supernatants from NS3-specific T cells coincubated with Lunet HCV+/HLA-A2+ (**Figure 5C**). In contrast, no statistical difference was observed in aspartate transaminase levels from the supernatant of NS5-specific TCR-T or mock non-transduced T cells when incubated with Lunet HCV+/HLA-A2+ and Lunet HCV+/HLA-A2- (**Figure 5F**), suggesting that reduced luciferase activity was based on the inhibition of HCV replication by IFNγ rather than by cytotoxicity.

Having verified T cell-mediated killing in the 2D assay, we moved to 3D cell culture conditions as setup for mimicking T cell-mediated cytotoxicity in a complex and tumor-like environment. The Huh-7/Lunet HCV replicon cells were formed as tumor spheroids (38) to assess the ability of TCR-T cells to mediate cytotoxic killing in a model that might be more predictive of *in vivo* efficacy.

To characterize the effects of TCR-T cells on the cell viability of tumor spheroids, we first examined the green fluorescent viable cells and spheroid integrity through image-based measurements using the S3 Incucyte live imaging and analysis system. The calcein-labeled cells were grown as single 3D spheroids in a 96-well round-bottom ultra-low attachment plate in the Incucyte. After 48 h, a single-cell suspension of Lunet HCV+ cells consistently formed spheroids that were spherically shaped and uniformly sized at basal time (0 h). Furthermore, for image-based quantification of cell viability in 3D cultures, spheroids cocultured with both NS3- or NS5-specific TCR-T cells were analyzed for cell viability in 3D cultures *via* real-time image-based quantification for 72 h. As illustrated in **Figure 5G**, after 48 h, the NS3-specific TCR-T cells markedly infiltrated the Lunet HCV+/HLA-A2+ spheroids, inducing a significant reduction on viable cells in a time-dependent manner (**Figure 5H**). Conversely, T cell infiltration and a significant reduction in cell viability were not observed in the spheroids incubated with NS5-specific TCR-T cells (**Figure 5I**). The analysis of Lunet HCV+/HLA-A2- spheroids cocultured with NS5-specific TCR-T cells revealed no impact in cell viability and no T cell infiltration (**Figure 5J**).

To further examine if cell viability reduction was related to apoptosis, we performed Annexin V staining followed by fluorescence-activated cell intensity measurement using Incucyte. Concurrently with the reduced cell viability, cell apoptosis, as indicated by Annexin V green fluorescent binding cells, dramatically increased in Lunet HCV+/HLA-A2+ spheroids following the addition of NS3-specific TCR-T cells compared to the control conditions. As shown in **Figure 5K**, Annexin V fluorescence intensity significantly increased in response to NS3-specific TCR-T cell incubation followed by 72 h (**Figure 5L**). In contrast, an increase in Annexin V green fluorescent signal was not observed in the spheroids incubated with NS5-specific TCR-T cells (**Figure 5M**). No significant increase in Annexin V green fluorescent signal was observed when NS3- or NS5-specific TCR-T cells were cocultured with spheroids derived from Lunet HCV+/HLA-A2-.

These results altogether reinforce the differences between NS3- and NS5-specific TCR-T cells in terms of cytotoxic activity: NS3-specific TCR-T cells are prone to induce the cytolysis of target cells, while NS5-specific TCR-T cells favor a non-cytotoxic mechanism. These findings support the previously described observations regarding NS3- and NS5-redirected T cells generated in a small scale by other methodologies, such as transient mRNA transfection or retroviral transduction (20, 29). Thus, our data indicate that our large-scale manufacturing of TCR-T cells preserves the original and distinct functional attributes of different TCR-T cell products, an important aspect to consider in order to develop a manufacturing protocol that can be adapted to multiple TCRs targeting different antigens.

## Discussion

ACT, as a therapeutic approach, holds great promise for cancer therapy and chronic viral diseases. However, these therapies also face challenges in terms of a standardized, reproducible, and economically feasible manufacturing process, where safety and efficacy have to be guaranteed within multiple regulatory frameworks. Genetically engineered T cells represent a complex biological living product, where quality depends not only on donor-to-donor variation but also on the manufacturing environment and the quality of complementary raw materials and reagents (39).

In this study, we reported for the first time the effectiveness of the lentiviral gene transfer approach to redirect human T cells with TCRs specific for two well-characterized human cytotoxic T cell epitopes with clinical relevance in hepatitis C infection (40–42). In our academic setting, we successfully generated TCR-T cells utilizing the CliniMACS Prodigy platform with performances comparable to other CAR products (16, 43–46). These findings add an important translational significance not only regarding a possible treatment option for chronic HCV and HCV-related liver cancer but notably also for other TCR-T cell therapies targeting different antigens with functionally diverse TCRs (47).

Our goal is to provide a simple, robust, and feasible manufacturing pipeline that can be used in academic settings to produce several TCR-T cell products maintaining the functional characteristics of each TCRs but providing reliable transduction efficiency, expansion, and phenotype.

In particular, we describe a rapid procedure as short as 8 days for the transduction and expansion of human T cells in a reproducible and scalable protocol for the clinical-grade manufacturing of TCR-T cells. Although the common expansion time for T cell products is around 14 days, it has been previously reported that shorter culture durations are associated with an improved anti-leukemic activity of CAR-T cells (48), and as long as the clinically relevant dose is achieved, the expansion can be limited to 7 (44) or 8 days (16). Since a clinically relevant dose is not defined yet for our product, we aimed to a production yield that could be used for a putative clinical dose of up to 5 × 10^6^ TCR+T cells/kg, which is similar to what has been used for other hepatitis-specific TCR-T cell products (30). Importantly, the final cell product specifications met the acceptable criteria similar to the clinical-grade products manufactured using established releasing criteria (4) for sterility, viability, phenotype, karyotype, transgene expression, and potency which attest the feasibility of our protocol in generating a GMP-compliant product.

One of our goals was to promote the expansion of CD8+ T cells without driving terminal differentiation. While most expansion protocols that utilize the CliniMACS Prodigy lead to a selective increase of the CD4+ T cell population (16, 44, 49), we wanted to obtain a T cell product that was the closest possible to a CD8/CD4 ratio of 1. The advantage of this is to provide more balanced cell products that could be utilized for several different TCRs, regardless of their co-receptor dependence. This approach is important in light of the well-known harnessing potential of cytotoxic CD8+ T cells in antitumor and antiviral response. The specific use of cytokines to supplement the cell culture media as well as the length of expansion might have possibly contributed to a less differentiated phenotype accompanied by a satisfactory number of CD8+ cells in the final product. Previous reports have shown that the addition of low-dose IL-2 in combination with IL-15 and IL-21 also resulted in the preferential outgrowth of CD8+ T cells in other T cell expansion models (50, 51).

In addition, we wanted to offer an example for a feasible set of T cell phenotypic and functional arrays. We designed a combined panel of surface memory/exhaustion markers such as CD62L, CD45RO, CCR7, and PD-1 and the transcription factor TCF1. We observed that the culture conditions favored the expression of cells with a central memory phenotype (Tcm and CD62L+/CD45RO+), which are the best candidates for a sustained *in vivo* response (52). In addition, the cells that expanded with our culturing conditions sustained the expression of TCF1 and CCR7, thereby facilitating the maintenance of a stem cell-like state. A less differentiated state would presumably be able to greatly persist *in vivo* and to exert an improved antitumor function (8).

As previously described, TCF1 is a key transcription factor of the Wnt signaling pathway, playing a pivotal role in peripheral T cell fate designation (53). Early after activation, a small subset of effector CD8+ T cells maintain TCF1 expression and give rise to central memory T cells (54, 55). Additionally, it has been shown that the presence of TCF1+ CD8+ T cells can be considered a requisite for a successful response to immune-checkpoint blockade following the administration of anti-PD-1 monoclonal antibodies (53). Indeed the antigen-specific cells that actually undergo a proliferative burst are “precursor exhausted T cells” (PD-1+ and TCF1+), whereas fully exhausted T cells (PD-1+ and TCF1-) cannot be rescued and exert any antitumor activity (56).

Despite the encouraging results in terms of the phenotype of the manufactured cells, we believe that the simple analysis of the commonly used surface markers might be difficult to interpret during the *ex vivo* cell expansion process in which the cells are artificially and strongly pushed to proliferate. Therefore, given the relevance of T cell phenotype for clinical response, an in-depth and thorough characterization of TCR-transduced T cells is eagerly needed to possibly identify a more reliable panel of optimal T cell attributes for adoptive cell therapy in cancer and chronic viral infections.

TCR-T cells were assessed for functionality and potency with multiple assays, spanning from peptide-loaded targets to HCV replicon cells, in 2D and 3D culturing conditions. Our results indicate that the cells were biologically functional and responded to the target cells in an antigen-specific manner. The observed polyfunctional profiles in response to the processed antigen support the results obtained with peptide-loaded T2 cells in which NS3-TCR activation stimulates IFNγ, IL-2, and TNFα in a dose-dependent manner, whereas NS5-specific CD8+ T cells predominantly expressed IFNγ, and no hepatocellular injury was observed. Notably, NS3-specific CD8+ cells appeared more polyfunctional than NS5-specific CD8+ cells. The ability of secreting pro-inflammatory cytokines not only is a common attribute of polyfunctional effector T lymphocytes but also has been correlated to a superior functional activity (57–60). These findings confirm the previously observed functional differences of the two TCR-T cells that were attributed mainly to a different TCR affinity (20, 29).

We introduced *in vitro* 2D and 3D potency assays for the development and manufacturing phases of engineered T cells. Firstly, our results show that the luciferase activity of Lunet HCV+/HLA-A2+, expressed as bioluminescence signals, decreased in a dose-dependent manner when incubated with NS3-specific TCR-T cells according to the E/T ratio. The NS5-specific TCR-T cells appeared to be less effective in controlling HCV replication, although a reduction of bioluminescence signal could still be observed in the experiments. Thus, our finding shows that both NS3- and NS5-specific TCR-T cells trigger an HLA-A2-restricted antiviral response against HCV replicon in hepatoma cell line in a 2D culture model. Moreover, we found an increase of aspartate transaminase levels in the supernatants from NS3-specific TCR-T cells and Lunet HCV+/HLA-A2+ cell cocultures, thus indicating a cytotoxic activity of NS3-specific TCR-T cells. These results reinforce the antiviral response and hepatotoxic potential of these cells as previously described by our group.

We used spheroids from our target hepatoma cell line coupled with an advanced live imaging system to follow the morphology, effector cell cytotoxicity, and tumor cell apoptosis (61). Our finding revealed that NS3-specific TCR-T cells infiltrate the tumor spheroids and significantly reduced cell viability in a time-dependent manner, whereas NS5-specific TCR-T cells did not affect cell viability and the spheroid’s structure. Furthermore, cell apoptosis, indicated by Annexin V, dramatically increased in the Lunet HCV+/HLA-A2+ spheroids after the addition of NS3-specific TCR-T cells compared to NS5-specific TCR-T cells. This finding further supports the idea that the difference in antigen specificity between the TCR-redirected T cells reflects also a different degree of cytotoxic potential as we previously observed. This type of rapid large-scale production can be applied to clinical development but can also be relevant to manufacture a large number of cells that can be used for pre-clinical evaluation in animal models (62, 63).

In conclusion the present study outlines the effectiveness of an optimized protocol for the manufacturing of TCR-T cells. Our strategy resulted in the generation of sufficient numbers of viable cells with a functional performance preserved after large-scale expansion. Importantly, the relatively rapid time of expansion and culture conditions allowed the achievement of the desired CD4/CD8 ratio in the final product together with the early differentiated memory phenotype of engineered cells. Overall, these data report a feasible and promising platform for the upcoming generation of antigen-specific TCR-T cell manufacturing.

## Data Availability Statement

The raw data supporting the conclusions of this article will be made available by the authors, without undue reservation.

## Author Contributions

DNS optimized protocols, designed and conducted the experiments, and wrote the manuscript. MC assisted with protocol development, flow cytometry analysis, and lentivirus production. GR conducted the experiments and drafted the manuscript. KH cloned TCRs in lentiviral vector. AKW assisted with live cell imaging assay development and analysis. PM and FG contributed to the final version of the manuscript. MM and LM edited the manuscript and provided scientific inputs. MSC supplied PBMCs from HCV chronic patient and cell lines used in the studies. VL provided the Lunet/HCV replicon cells. MS provided study oversight. MB obtained funding and assisted with the flow cytometry panel design. AP provided study oversight, obtained funding for the project, and assisted with drafting of the manuscript. All authors contributed to the article and approved the submitted version.

## Funding

This study was supported by the Sjöberg Foundation (AP), Region Stockholm Centrum För Innovativ Medicin (AP, MB, and MS), Svenska Läkaresällskapet (AP), Cancerfonden (AP, MB, and MS), Ruth och Richard Julins Stiftelse (AP), Vinnova project CAMP (contract no. 2017-02130) (MS), the EU-funded project ERINHA (MS), Swedish Science Council (MB and MS) and Region Stockholm ALF grant (MS), and the Deutsche Forschungsgemeinschaft (TRR179, 272983813) (VL).

## Conflict of Interest

The authors declare that the research was conducted in the absence of any commercial or financial relationships that could be construed as a potential conflict of interest.

## Publisher’s Note

All claims expressed in this article are solely those of the authors and do not necessarily represent those of their affiliated organizations, or those of the publisher, the editors and the reviewers. Any product that may be evaluated in this article, or claim that may be made by its manufacturer, is not guaranteed or endorsed by the publisher.
