# Supplementary material for: Process Development for Adoptive Cell Therapy in Academia: A Pipeline for Clinical-Scale Manufacturing of Multiple TCR-T Cell Products
Source: Front Immunol. 2022 Jun 16;13:896242. doi: 10.3389/fimmu.2022.896242 (PMC9243500; doi:10.3389/fimmu.2022.896242)
Supplement: Supplementary file 1 [file Image_1.pdf]

Supplementary Material

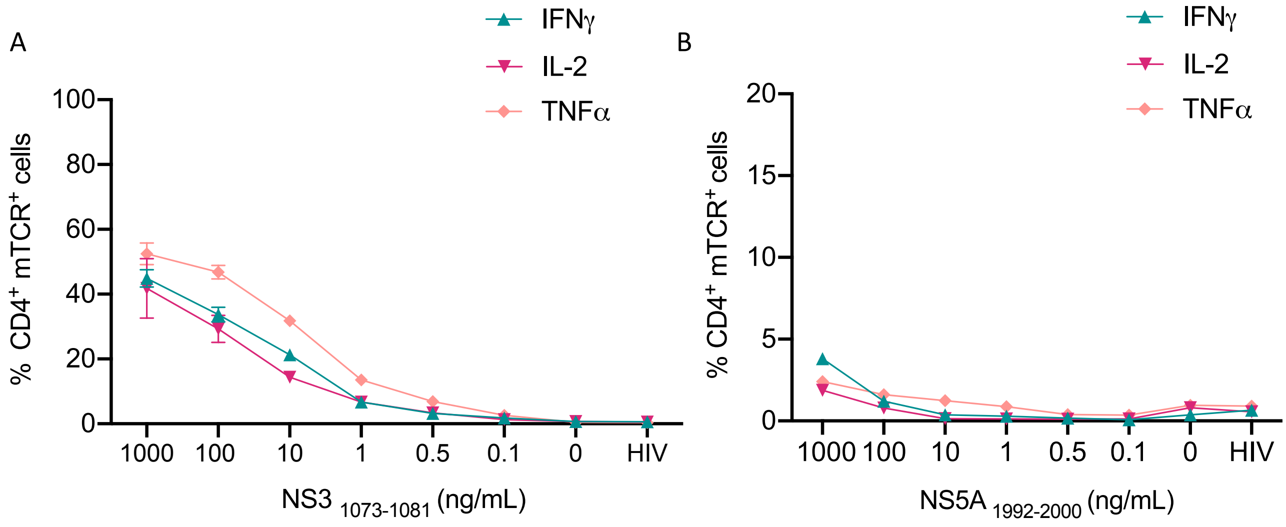

Supplementary figure 1: HCV-specific responses assessed by flow cytometry analysis for intracellular content of IFNγ, TNFα and IL-2 in CD4+ transduced T cells following 6h of coculture of NS31073–1081 (A) or NS51992–2000 (B) peptide-loaded T2 cells with indicated NS3- or NS5-specific TCR-T cells manufactured in CliniMacs Prodigy. HIV peptide-loaded T2 cells and non-stimulated T cells were included as negative controls in the coculture experiments performed in triplicate wells.

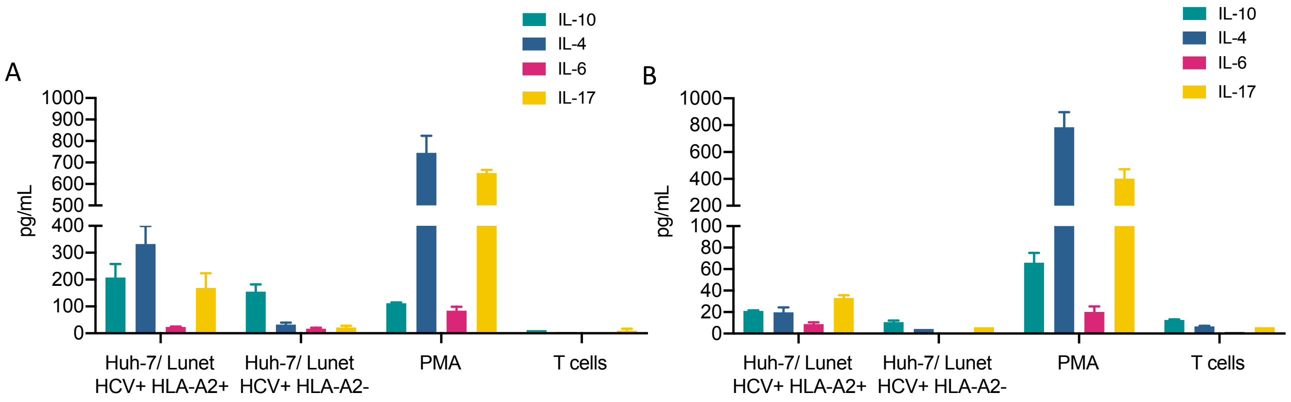

Supplementary figure 2: Cytokine production of TCR-T cells co-cultured with Huh-7/Lunet HCV replicon target cells measured by Milliplex map human high sensitivity kit. Supernatants were harvested after 24h and concentrations of human IL-6, IL-10, IL-17, IL-4 were evaluated in undiluted samples of NS3-specific TCR-T cells (A) or NS5-specific TCR-T cells (B). Huh-7/Lunet HCV replicon cells that were not engineered to express HLA-A2 (Lunet HCV+/HLA-A2-) as well as non-stimulated T cells were used as negative controls in the experiments performed with technical triplicates. Results shown as mean ± SD for three independent healthy donors.
